# Oxidative Stress in Canine Histiocytic Sarcoma Cells Induced by an Infection with Canine Distemper Virus Led to a Dysregulation of HIF-1α Downstream Pathway Resulting in a Reduced Expression of VEGF-B In Vitro

**DOI:** 10.3390/v12020200

**Published:** 2020-02-11

**Authors:** Federico Armando, Matteo Gambini, Attilio Corradi, Chiara Giudice, Vanessa Maria Pfankuche, Graham Brogden, Friederike Attig, Maren von Köckritz-Blickwede, Wolfgang Baumgärtner, Christina Puff

**Affiliations:** 1 Department of Pathology, University of Veterinary Medicine Hannover, Bünteweg 17, 30559 Hannover, Germany; federico.armando@unipr.it (F.A.); matteo.gambini@unimi.it (M.G.); vanessa.pfankuche@tiho-hannover.de (V.M.P.); friederike.attig@tiho-hannover.de (F.A.); christina.puff@tiho-hannover.de (C.P.); 2 Department of Veterinary Medicine, Pathology Unit, University of Parma, Strada del Taglio 10, 43126 Parma, Italy; attilio.corradi@unipr.it; 3 Dipartimento di Medicina Veterinaria (DIMEVET), Universitá degli Studi di Milano, Via dell‘Universitá 6, 26900 Lodi, Italy; chiara.giudice@unimi.it; 4 Center for Systems Neuroscience, University of Veterinary Medicine Hannover, 30559 Hannover, Germany; 5 Department of Physiological Chemistry, University of Veterinary Medicine Hannover, Bünteweg 17, 30559 Hannover, Germany; Graham.brogden@twincore.de (G.B.); maren.von.koeckritz-blickwede@tiho-hannover.de (M.v.K.-B.); 6 Research Center for Emerging Infections and Zoonoses (RIZ), University of Veterinary Medicine Hannover; Bünteweg 17, 30559 Hannover, Germany

**Keywords:** angiogenesis, canine distemper virus, canine histiocytic sarcoma, DH82, HIF-1α, oxidative stress, VEGF-B, viral oncolysis

## Abstract

Histiocytic sarcomas represent malignant tumors which require new treatment strategies. Canine distemper virus (CDV) is a promising candidate due to its oncolytic features reported in a canine histiocytic sarcoma cell line (DH82 cells). Interestingly, the underlying mechanism might include a dysregulation of angiogenesis. Based on these findings, the aim of the present study was to investigate the impact of a persistent CDV-infection on oxidative stress mediated changes in the expression of hypoxia-inducible factor (HIF)-1α and its angiogenic downstream pathway in DH82 cells in vitro. Microarray data analysis, immunofluorescence for 8-hydroxyguanosine, superoxide dismutase 2 and catalase, and flow cytometry for oxidative burst displayed an increased oxidative stress in persistently CDV-infected DH82 cells (DH82Ond pi) compared to controls. The HIF-1α expression in DH82Ond pi increased, as demonstrated by Western blot, and showed an unexpected, often sub-membranous distribution, as shown by immunofluorescence and immunoelectron microscopy. Furthermore, microarray data analysis and immunofluorescence confirmed a reduced expression of VEGF-B in DH82Ond pi compared to controls. In summary, these results suggest a reduced activation of the HIF-1α angiogenic downstream pathway in DH82Ond pi cells in vitro, most likely due to an excessive, unusually localized, and non-functional expression of HIF-1α triggered by a CDV-induced increased oxidative stress.

## 1. Introduction

Neoplastic diseases are one of the major causes of death in humans and domestic animals due to disappointing results of many conventional therapies [1,2]. Therefore, viral oncolysis represents an interesting potential new option in human as well as veterinary medicine [3,4,5]. Interestingly, viruses from different families, including members of the *Paramyxoviridae* (canine distemper virus, measles virus and Newcastle disease virus), *Poxviridae* (vacciniavirus), *Reoviridae* (reovirus serotype 3 Dearing), *Adenoviridae* (adenovirus Onyx-015 and H101), *Orthomyxoviridae* (influenza virus), *Herpesviridae* (herpes simplex virus type 1), *Picornaviridae* (coxsackievirus) and *Rhabdoviridae* (vesicular stomatitis virus) possess oncolytic properties [6,7,8].

Canine distemper virus represents a *Morbillivirus* closely related to human measles virus [9], with the latter already described as a promising oncolytic virus in human medicine that has reached the phase of clinical trials [10]. Similarly, the attenuated Onderstepoort vaccine strain of canine distemper virus (CDV-Ond) represents a potential oncolytic virus for the treatment of canine histiocytic sarcomas [11,12].

Canine histiocytic sarcomas are malignant tumors with poor prognosis and limited therapeutic options [13,14] which originate from interstitial dendritic cells or from macrophages [15,16,17,18]. Since its establishment in 1988 [19], a canine histiocytic sarcoma cell line (DH82 cells) has been commercially available. DH82 cells can be infected by CDV-Ond [12], and have been reported as a promising model for the investigation of viral oncolysis [8,20,21]. Specifically, acute infection of DH82 cells with CDV-Ond in vitro resulted in a prominent cell death at 12 days post infection [12], followed by establishment of persistent infection in tumor cells surviving the acute lytic phase [11]. In this context, subcutaneous xenotransplantion of persistently CDV-Ond infected DH82 cells resulted in a total regression of neoplasms in a mouse model [11]. This promising observation was assumed to be related to a decreased vascularization of the transplants [11], with the underlying mechanisms not fully understood so far. Therefore, additional investigations using persistently CDV-infected DH82 cells might represent a promising model to study virus-induced alterations of cancer hallmarks [22] and of the tumor microenvironment [23] avoiding the confounding effects correlated with ongoing virus-induced cytopathogenic tumor cell death associated with the acute infection [12]. Indeed, as reviewed by Lapp et al. [8], viral oncolysis mechanisms can be distinguished between primary (i.e., direct virus-induced cytolysis and/or apoptosis) and secondary ones. The latter include a wide range of events leading to tumor cell death, such as modulation of the antiviral and antitumoral immune response, changes in the organization of the tumor-associated extracellular matrix, and alterations of the tumor-associated vasculature and angiogenesis [3,4,7,8,11,23,24,25,26].

Specifically, a reduced vascularization of neoplasms often leads to intratumoral hypoxia [27] associated with modifications especially of intracellular pathways connected with reactive oxygen species (ROS) production and scavenging. ROS are highly chemically reactive molecules that can induce damage to cellular macromolecules such as nucleic acid and lipids, when they outnumber scavenging systems [28,29,30]. CDV infection can increase ROS production and ROS-induced damage in vitro and in vivo as shown for spontaneous CDV infection in dogs [31,32,33,34,35]. Furthermore, CDV can induce an accumulation of viral glycoproteins in the endoplasmic reticulum (ER) of Vero cells and primary rat neurons, resulting in increased endoplasmic reticulum stress [36], which has been reported as associated with an increased ROS production [37]. Nevertheless, ROS are physiologically involved in a plethora of different intracellular signaling pathways [29,30], and play a key role in multiple hallmarks of cancer [38].

Hypoxia-inducible factor 1-alpha (HIF-1α) is a transcription factor that after translocation from the cytoplasm to the nucleus, forms a heterodimer with hypoxia-inducible factor 1-beta (HIF-1β), which binds to specific DNA sequences known as hypoxia response elements (HREs) [39,40]. This event induces the expression of numerous genes involved in different cellular responses such as angiogenesis [40,41], which is driven by several growth factors, including members of the vascular endothelial growth factor (VEGF) family.

Hypoxia, and to a lesser extent ROS, represent the most important stimuli for HIF-1α stabilization and nuclear translocation [39,42]. During normoxia and redox homeostatic state [28], HIF-1α is localized within the cytoplasm and is rapidly degraded by the proteasome after hydroxylation by prolyl hydroxylases (PHDs) and subsequent ubiquitination by the von Hippel-Lindau protein (VHL) [39,40,42]. In this context, hypoxia and ROS directly down-regulate the activity of PHDs and VHL [42], playing therefore a key role in the inhibition of the overall HIF-1α degradation.

In consideration of the above, the hypothesis of the present study was that a persistent CDV-Ond infection of DH82 cells induces oxidative stress followed by a massive inhibition of HIF-1α degrading pathways. This in turn leads to cytoplasmic, non-functional accumulation of HIF-1α, which is associated with a reduced expression of HIF-1α downstream targets, such as VEGF-B.

Based on the aforementioned hypothesis, the aim of the present in vitro study was to demonstrate that histiocytic sarcoma cells (DH82 cells) persistently infected with CDV-Ond show: (1) an increased oxidative stress status, (2) an increased HIF-1α protein expression, (3) an unusual intracellular distribution of HIF-1α, and (4) a reduced expression of HIF-1α downstream targets, with a special focus on VEGF-B.

## 2. Materials and Methods

### 2.1. Cell Culture and Production of Cell Pellets

Non-infected DH82 cells obtained from the European Collection of Authenticated Cell Cultures (ECACC No. 94062922), and DH82 cells persistently infected with CDV-Ond (DH82Ond pi) that were established as formerly described [20], were cultured according to standard procedures as previously reported [11]. Briefly, cells were cultured in minimal essential medium (MEM) with Earle’s salts (PAA, Cölbe, Germany) supplemented with 10% fetal calf serum (PAA), 1% penicillin/streptomycin (PAA), and 1% non-essential amino acids (Sigma-Aldrich, Taufkirchen, Germany). Culture flasks were kept at 37 °C in the presence of 5% CO_2_ in a water saturated atmosphere.

Five formalin-fixed paraffin embedded (FFPE) cell pellets of non-infected DH82 cells and 5 of DH82Ond pi cells were produced as previously described [43]. Briefly, cells were scraped and centrifuged at 250xg for 10 min at 4 °C. Afterwards, the supernatant was removed, cells were washed in PBS and centrifuged again. Following a second wash and centrifugation step, the pellet was fixed in 1.5 mL of 10% non-buffered formalin overnight at 4 °C, and processed for routine paraffin embedding.

### 2.2. Microarray Data Analysis Using a Manually Generated List of Gene Symbols Related to ROS Production and Scavenging, ER Stress and HIF-1α Pathway

In a hypothesis-driven approach, an online available microarray data set of quadruplicates of non-infected DH82 and DH82Ond pi cells (ArrayExpress; http://www.ebi.ac.uk/arrayexpress; accession number E-MTAB-3942 [11,44]) was investigated for differentially expressed genes related to ROS production and scavenging, ER-stress and HIF-1α pathway, with a special focus on the angiogenic downstream targets of the latter. This choice was justified by the results of the functional profiling of the same dataset obtained in a previous study, highlighting a down-regulation of the expression of some of the genes correlated with angiogenesis [11]. Therefore, in the current work, a list of human and murine genes and proteins was manually generated according to the literature [29,31,36,37,39,40,41,42,45,46] and translated into canine orthologous gene symbols using the web-based HGNC database (HGNC Database, HUGO Gene Nomenclature Committee (HGNC), European Molecular Biology Laboratory, European Bioinformatics Institute (EMBL-EBI), Wellcome Genome Campus, Hinxton, Cambridge CB10 1SD, United Kingdom, www.genenames.org [47]). After filtration, the raw expression data of the selected genes were compared between non-infected DH82 and DH82Ond pi cells, employing multiple pairwise nonparametric Mann–Whitney U-tests. Statistical analysis was performed with SAS Enterprise Guide (SAS version 9.3; SAS Institute Inc, Cary, NC, USA). Differential expression was defined as the combination of a fold change (FC) filter (FC ≥ 1.5 or ≤ −1.5) and of a statistical significance filter (Mann–Whitney U-test; *p* ≤ 0.05) [48]. To facilitate the interpretation of results, each gene symbol was assigned to at least one of the following functional groups on the basis of the function(s) carried out by its corresponding protein(s): ROS production; ROS scavenging; ER stress; HIF-1α activation, transcriptional activity and regulation; HIF-1α angiogenic downstream pathway.

### 2.3. Immunofluorescence and Statistical Analysis

Immunofluorescence was performed on FFPE pellets of non-infected and persistently CDV infected DH82 cells as previously described with minor variations [11,49]. Briefly, sections were deparaffinized, rehydrated through graded alcohol and pre-treated for antigen retrieval. Following blocking of unspecific bindings, sections were incubated with primary antibodies for 90 min at room temperature. After 60 min of incubation with the secondary antibody, nuclei were stained with Bisbenzimide (Sigma-Aldrich Chemie GmbH, Taufkirchen, Germany), and the slides were mounted with Dako Flourescence Mounting Medium (Dako North America, Inc., Carpinteria, CA, USA). Each reaction was carried out with corresponding positive controls (Table 1). For negative controls, the first antibody was replaced with rabbit serum, Balb/c ascitic fluid, or goat serum, respectively at corresponding protein concentrations. To verify the persistent infection status of DH82Ond pi cells (which was set as corresponding to a rate of >95% infected cells), an immunolabeling with an anti-CDV nucleoprotein (CDV-NP) antibody (clone D110; kindly provided by Prof. A. Zurbriggen, University of Bern, Switzerland) was performed. Furthermore, pellets were stained with antibodies directed against 8-hydroxyguanosine/8-hydroxydeoxyguanosine (8OHG/8OHdG, in the following paragraphs simply referred to as 8OHdG), a marker of ROS-damaged RNA or DNA [31]; superoxide dismutase 2 (SOD2) and catalase (CAT), two ROS scavengers; HIF-1α, a transcription factor; wheat germ agglutinin (WGA), a cell membrane marker; CD63, directed against tetraspanin-30 expressed on exosome membranes; and GM-130, a marker for Golgi apparatus. All details regarding the antibodies used are listed in Table 1.

For CDV-NP, 8OHdG, SOD2, CAT, HIF-1α, and VEGF-B, the percentage of immunopositive cells for each group (non-infected DH82 cells and DH82Ond pi cells) was assessed manually by counting 5 evenly distributed fields per pellet at a 400x magnification using an inverted fluorescence microscope (Olympus IX-70, Olympus Optical Co. GmbH, Hamburg, Germany) equipped with a Olympus DP72 camera and Olympus cellSens standard software version 2.3. Additionally, for HIF-1α the intracellular protein distribution was assessed and calculated as percentage of cells immunopositive within the nucleus, cytoplasm and membrane. For each marker, after calculation of the median percentage of immunopositive cells per pellet, the normality of distribution of the data referring to non-infected and DH82Ond pi cells was evaluated with the Shapiro-Wilk test and followed by the Mann-Whitney U test for pairwise comparison. The difference of the intracellular distribution of HIF-1α immunopositivity within each group of cells was analyzed with the Kruskall-Wallis test with post-hoc Dunn’s test. Statistical significance for each analysis was set at p-value ≤ 0.05. All statistical analyses were performed with GraphPad Prism version 8.0.1 for Windows (GraphPad Software, La Jolla, CA, USA, www.graphpad.com).

### 2.4. Determination of Oxidative Burst by Flow Cytometry

Non-infected and persistently CDV-Ond infected DH82 cells were treated with 2´,7´-dichlorofluorosceindiacetate (DCF, final concentration of 10 μM, Sigma Aldrich, D6883) at 37 °C and 5% CO_2_ for 20 min. Flow cytometer (Attune^®^ NxT Acoustic Focusing; laser 488 nm (50 mW), filter BL-1  =  530/30) analysis was performed measuring mean green fluorescence intensity (X-Mean of BL-1) as relative ROS production. Respective background controls without DCF were included in all assays. Threshold was adjusted to unstained cells to remove background. Green fluorescence intensity (FITC) of all cells (percentage of positive cells) was recorded by flow cytometry as relative measure of ROS production. The following settings were used: acquisition volume of 100 µL/min, stop at 10,000 events on all counts; instrument settings: FSC 80, SSC 320 BL1 310 (FITC).

For quantification of the percentage of positive cells, doublets were excluded by FCS-A versus FSC-H gating (see Supplementary Data) and only FL-1-positive cells (Gate 2) of all singlet cells (Gate 1) were quantified. Statistical analyses of measurements were performed with GraphPad Prism version 8.0.1 for Windows (GraphPad Software, La Jolla California USA, www.graphpad.com) using unpaired *t*-tests.

### 2.5. Immunoelectron Microscopy

To evaluate in more detail the intracellular localization of HIF-1α within DH82Ond pi cells, immunoelectron microscopy was performed using a 10% neutral buffered formalin fixed cell pellet as previously described [50]. Ultrathin sections of LR-White embedded samples were immunolabeled with an anti-HIF-1α antibody (1:500 dilution; Novus biologicals) followed by a goat anti-rabbit IgG 10 nm immunogold conjugated secondary antibody (BBI Solutions, Crumlin, United Kingdom). Samples were further evaluated using a transmission electron microscope (EM 10A, Carl Zeiss Microscopy GmbH, Jena, Germany) equipped with a 2K-CCD-Camera (TRS) and using Image SP professional software.

### 2.6. Laser Scanning Confocal Microscopy

The intracellular HIF-1α distribution was analyzed by double-labeling immunofluorescence (DL-IF). Therefore, HIF-1α was combined with WGA as a marker for the cell membrane, CD63 as an exosomal marker, GM-130 as a marker for the Golgi apparatus, and CDV-NP. The evaluation was performed using a Leica TCS SP5 II fluorescence microscope (Leica Microsystems, Bensheim, Germany) with a conventional galvanometer scanner of the Leica SP5 II tandem scanning system and the Leica Application Suite Advanced Fluorescent Lite 2.0.2 build 2038 (Leica, Biberach, Germany). Settings were adjusted using respective control antibodies. Images were analyzed using Leica LAS AF software (version 2.7.3).

### 2.7. Immunoblotting

Cell lysates were prepared by freezing and thawing in 1 mL NP-40 buffer (50mM Tris-HCl, 150 mM NaCl, 1% NP-40, 5 mM EDTA) with 50 µL protease inhibitor cocktail (1.48 µM Antipain dihydrochloride, 0.768 µM Aprotinin, 10.51 µM Leupeptin, 1.46 µM Pepstatin A in DMSO, 1 mM PMSF, 50 µg/mL Trypsin inhibitor T9128) at pH 8.0 (all reagents from Sigma-Aldrich, St. Louis, USA). Samples were analyzed by SDS-PAGE on 8% gels and subsequently transferred to a Polyvinylidene fluoride (PVDF) membrane as described previously [51]. Immunoblotting was performed using a polyclonal anti-HIF-1α (0.75 µg/mL, Cayman, Ann Arbor, USA) and a monoclonal anti-β-actin (0.2 µL/mL, Santa Cruz, Dallas, USA) antibody, respectively. A polyclonal IgG antibody from rabbit serum served as a negative control (2 µg/mL, Sigma-Aldrich, St. Louis, USA). Secondary anti-rabbit or anti-mouse antibodies conjugated to horseradish peroxidase were used (0.2 µg/mL, ThermoScientific, Schwerte, Germany). Protein bands were visualized using SuperSignal™ West Femto maximum sensitivity western blot chemiluminescence substrate (ThermoScientific, Schwerte, Germany) and a ChemiDoc MP Imaging System (Bio-Rad, Hercules, CA, USA). Quantification was performed densitometrically. Obtained results for HIF-1α were displayed as a ratio with the corresponding amount of β-actin. Statistical analyses of obtained ratios were performed with GraphPad Prism version 8.0.1 for Windows (GraphPad Software, La Jolla, CA, USA, www.graphpad.com) using unpaired *t*-tests.

## 3. Results

### 3.1. Persistent CDV Infection of DH82cells Leads to an Increased Level of Intracellular ROS Associated with Increased Catalase and Superoxide Dismutase 2 Protein Expression

The infection status of DH82Ond pi cells was assessed via immunofluorescence staining for CDV-NP (Supplementary Figure S1). While immunoreactivity for CDV-NP of non-infected DH82 cell pellets was negative in all cells, DH82Ond pi cell pellets showed a median percentage of 99.65% (range: 99.05–100.00%) infected cells (Supplementary Table S1).

On a molecular level, a manually generated list of 235 canine gene symbols associated with ROS production and scavenging, ER stress and the HIF-1α pathway was analyzed using a microarray dataset of DH82 and DH82Ond pi cells. This investigation resulted in a list of 230 genes present within the available data set (Supplementary Table S2). Using the combination of a statistical significance filter (Mann–Whitney U-test; *p* ≤ 0.05) and a fold change (FC) filter (FC ≥ 1.5 or ≤ −1.5), 57 genes were differentially expressed. Specifically, 31 canine genes showed a down-regulation, whereas 26 genes were up-regulated (Table 2).

When specifically analyzed according to the functional grouping, 12 genes related to ROS production were up-regulated, while nine were down-regulated (Table 2). Among the group of genes related to ROS scavenging, five genes were up- and five were down-regulated (Table 2). Specifically, neutrophil cytosolic factor 4 (*NCF4*) and thioredoxin interacting protein (*TXNIP*), belonging to ROS production and ROS scavenging functional groups, respectively, were the two most markedly up-regulated genes among the entire set examined. Taken together, these findings should be cautiously interpreted as an increased transcription of genes which corresponding proteins are involved in increasing intracellular oxidative stress [29,41,52,53]. Among the group of genes related to ER-stress (partially overlapping with both ROS production and ROS scavenging functional groups), 12 genes were up-regulated while 14 were down-regulated. Specifically, among the genes included in the ER stress functional group, the xanthine dehydrogenase (*XDH*) was up-regulated, while among down-regulated genes were included 3 (*PDIA3*, *PDIA4* and *PDIA6*) out of 4 genes related to protein disulphide isomerases, one (*ERO1L*) out of two genes related to endoplasmic reticulum oxidoreductines, and two (*CANX* and *DDIT3*) out of three genes previously related to ER-stress induced by acute infection with CDV [36]. Taken together, these results can be cautiously interpreted as indicative of a reduced transcription of genes that are reported to correlate with ER-stress [36,54,55,56,57].

**Figure 1:**
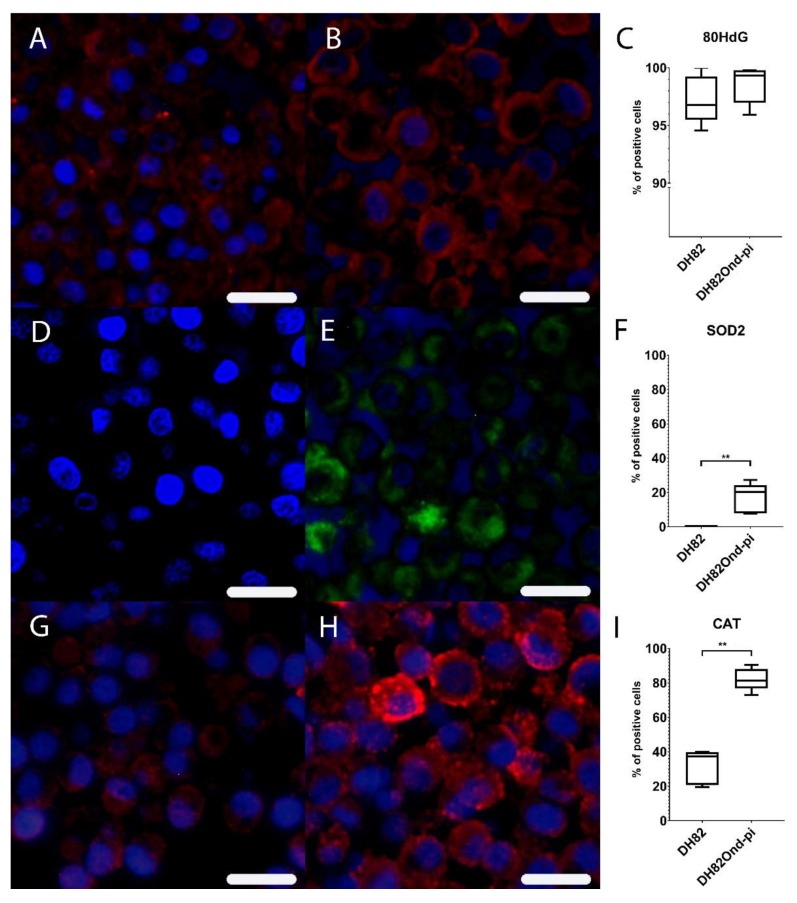
Immunofluorescence analysis revealed a lower expression of markers associated with oxidative stress in non-infected (**A**,**D**,**G**) compared to persistently canine distemper virus (CDV) infected (**B**,**E**,**H**) DH82 cells. Staining for 8OHdG (Cy3, red) and bisbenzimide (nuclei, blue) revealed a similar expression in non-infected (**A**) and persistently CDV-infected (**B**) DH82 cells as graphically shown in (**C**). Staining for superoxide dismutase (Cy2, green) and bisbenzimide (nuclei, blue) showed a significantly lower expression in non-infected (**D**) compared to persistently CDV-infected DH82 (**E**) cells as graphically depicted in (**F**). Staining for catalase (Cy3, red) and bisbenzimide (nuclei, blue) demonstrated a significantly lower expression in non-infected (**G**) compared to persistently CDV-infected (**H**) DH82 cells as graphically shown in (**I**). Bar = 20µm. (**C**), (**F**) and (**I**) display box and whisker plots with median values, quartiles and maximum and minimum values. Significant differences (*p* ≤ 0.05, Mann–Whitney U-test) are labeled by asterisks (** *p* ≤ 0.01).

The hypothesized increased oxidative stress in DH82Ond pi cells compared to non-infected DH82 cells was further investigated by means of immunoreactivity for 8OHdG, SOD2 and CAT, as displayed in Figure 1, as well as by determination of oxidative burst by flow cytometry.

Immunofluorescence for 8OHdG lacked a significant difference (*p* = 0.5476) in the percentage of positive cells between non-infected (median = 96.80%, range: 94.58–100.00%) and DH82Ond pi pellets (median = 99.33%, range: 95.94–99.79%) (Supplementary Table S1). Immunofluorescence for SOD2 displayed a significantly (*p* = 0.0079) increased percentage of positive cells in DH82Ond pi pellets (median = 20.39%, range: 7.75–27.30%) compared to non-infected DH82 pellets (median = 0.00%, range: 0.00%–0.47%) (Supplementary Table S1). Immunofluorescence for CAT revealed a significantly (*p* = 0.0079) increased percentage of positive cells in DH82Ond pi pellets (median = 81.29%, range: 72.92%–90.58%) compared to non-infected DH82 pellets (median = 37.27%, range: 19.61%–39.94%) (Supplementary Table S1).

**Figure 2:**
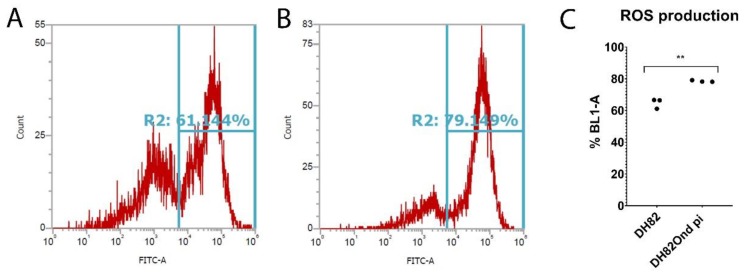
Determination of oxidative burst by fluorescence activated cells sorting (FACS) in non-infected (**A**) and persistently canine distemper virus (CDV) infected (**B**) DH82 cells. The percentage of cells positive for ROS-formation was measured by flow cytometry (BL-1) using a DCF fluorescence probe. (**C**) BL-1 positive cells revealed a significantly increased ROS production among persistently CDV-infected DH82 cells compared to non-infected controls. All data are shown as dot plots with means ± standard error of mean. Significant differences (*p* ≤ 0.05, unpaired *t*-test) are labeled by asterisks (** *p* ≤ 0.01).

The determination of oxidative burst by flow cytometry demonstrated a significantly (*p* = 0.0017) increased ROS production among DH82Ond pi cells compared to non-infected DH82 cells (Figure 2).

Despite a lack of difference in ROS-induced nucleic acid damage as determined by immunofluorescence of 8OHdG, these results are collectively indicative of an increased oxidative stress in DH82Ond pi cells compared to non-infected DH82 cells, which might lead to an increased level of HIF-1α and subsequently to an inhibition of its degradation.

### 3.2. DH82Ond pi Are Characterized by an Increased HIF-1α Protein Expression Associated with an Altered Intracellular Distribution

Among the gene symbols referring to the functional group “HIF-1α activation, transcriptional activity and regulation”, three out of 15 genes were down-regulated (Table 2). Specifically, down-regulated gene symbols were those referring to two (*ENGL1* and *ENGL3*) out of three prolyl hydroxylases and to von Hippel-Lindau (*VHL*) protein, while HIF-1α gene symbol (*HIF1A*) did not show any significant change (Supplementary Table S2).

**Figure 3:**
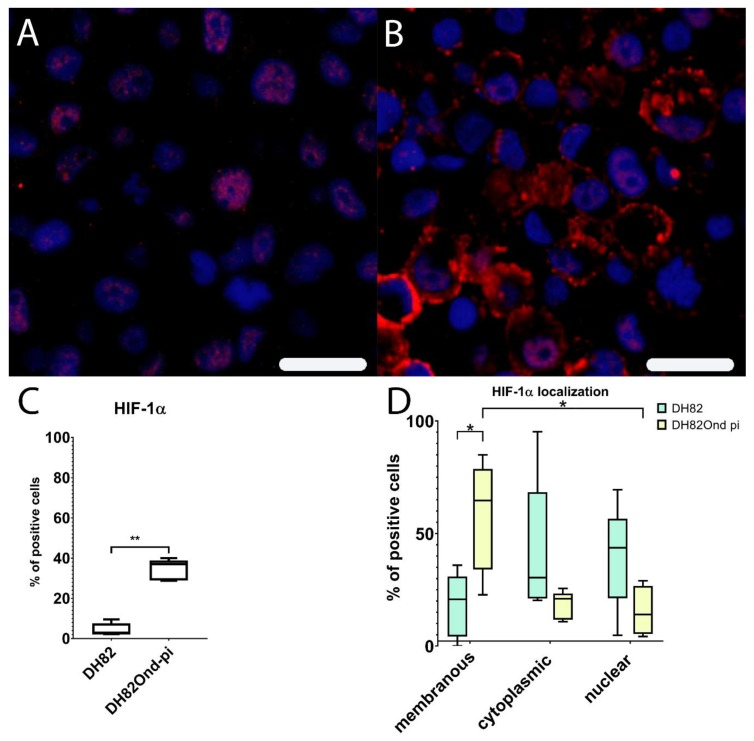
Immunofluorescence analysis for HIF-1α expression (Cy3, red; bisbenzimide, blue, nuclei) reveals a lower membranous expression in non-infected (**A**) compared to persistently canine distemper virus (CDV) infected (**B**) DH82 cells. Non-infected DH82 cells frequently displayed a nuclear immunolabeling (**A**) whereas a frequent membrane-associated staining was observed in persistently CDV-infected DH82 cells (**B**). Bar = 20µm. HIF-1α shows a significantly increased percentage of positive cells in persistently CDV-infected DH82 cells compared to non-infected controls (**C**). (**D**) Within non-infected DH82 cells, HIF-1α was present within nucleus and cytoplasm without significant differences between the localizations. In contrast, persistently CDV-infected DH82 cells displayed a significantly higher membranous HIF-1α expression compared to nuclear (*p* = 0.0486) but not to cytoplasmic (*p* = 0.0710) localizations. Additionally, the membranous immunopositivity for HIF-1α in persistently CDV-Ond infected DH82 cells was significantly higher compared to the corresponding localization in non-infected controls. Box and whisker plots display median and quartiles with maximum and minimum values. Significant differences (*p* ≤ 0.05, Mann–Whitney U-test (**C,D**) and Kruskall-Wallis test with post-hoc Dunn’s test (**D**)) are labeled by asterisks (* *p* ≤ 0.05 and ** *p* ≤ 0.01).

Immunoreactivity for HIF-1α revealed a significant (*p* = 0.0079) higher percentage of positive DH82Ond pi cells (median = 36.95%, range 28.83%–39.99%) (Supplementary Table S1) compared to non-infected DH82 cells (median = 2.53%, range: 2.24%–9.51%), as shown in Figure 3. In non-infected DH82 cells, HIF-1α was mainly expressed within nucleus (median = 43.69%, range: 4.76%–69.49%) and cytoplasm (median = 30.38%, range: 20.31%–95.24%) and only to a lesser extent in the membrane (median: 20.75%, range: 0.00%–35.94%), without significant differences (*p* ranging from 0.1980 to >0.9999) between the three localizations. Interestingly, DH82Ond pi cells displayed a significantly higher HIF-1α expression in the membrane (Figure 3) compared to nuclear (*p* = 0.0486; membrane median = 64.74%, membrane range: 22.80%–85.02%; nuclear median = 14.06%, nuclear range: 4.20%–29.05%) but not to cytoplasmic localizations (*p* = 0.0710; cytoplasm median = 21.01%, cytoplasm range: 10.78%–25.58%). Additionally, the membranous immunopositivity for HIF-1α in DH82Ond pi cells was significantly (*p* = 0.0317) higher when compared to the corresponding localization in non-infected DH82 cells (Supplementary Table S1).

**Figure 4:**
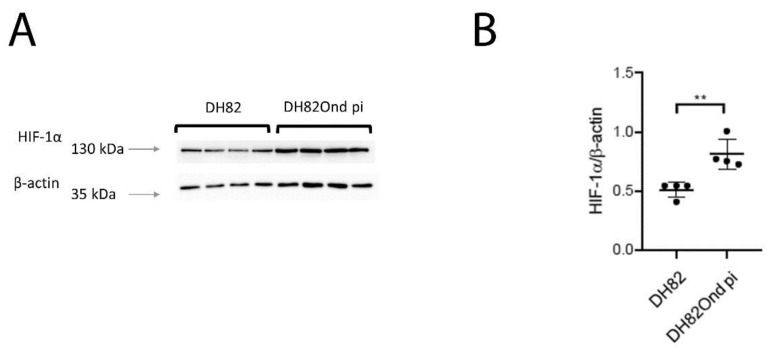
Immunoblotting with anti-HIF-1α and anti-β-actin antibodies revealed a single band of approximately 130 kDa and 43 kDa, respectively, when compared to the corresponding marker lengths of 130 kDa and 35 kDa (arrows, **A**). (**B**) Band intensities and sizes of both HIF-1α and beta-actin were quantified and their ratio determined, revealing a significant increase of HIF-1α in persistently canine distemper virus (CDV)-infected DH82 cells compared to non-infected controls. Dot plots display means and standard deviation. Significant differences (*p* ≤ 0.05, unpaired *t*-test.) are labeled by asterisks (** *p* ≤ 0.01).

HIF-1 α immunoblotting confirmed the significantly increased protein expression (*p* = 0.0027) in DH82Ond pi cells when compared to the non-infected DH82 cells (Figure 4).

Summarized, these results are indicative of an increased level of HIF-1α in DH82Ond pi, which is most likely due to a decreased cytoplasmic degradation. To further characterize the intracellular localization of HIF-1α, immunoelectron microscopy and laser scanning confocal microscopical analysis of double stainings were performed.

### 3.3. DH82Ond pi Show an Unusual Mainly Sub-Membranous Distribution of HIF-1α

**Figure 5:**
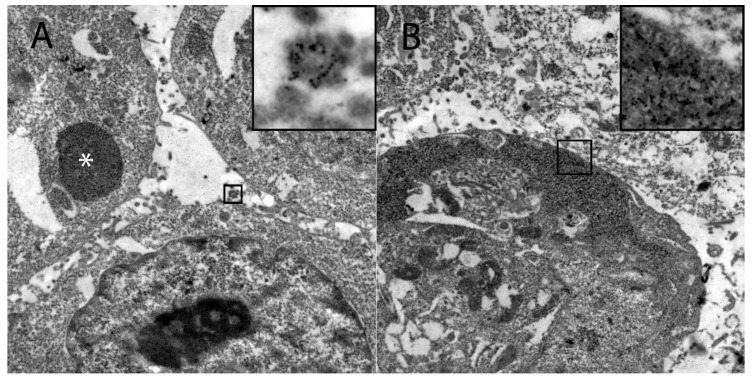
Demonstration of the intracellular HIF-1α localization in persistently canine distemper virus infected DH82 cells as determined by immunoelectron microscopy. (**A**) HIF-1α was found within variably sized, round, moderately to highly electrondense vesicles (insert) and in large moderately electrondense vacuoles (*). Additionally, HIF-1α was detected often in the sub-membranous area of the cytoplasm (insert; **B**). Magnification 9000×.

Ultrastructural investigation of DH82Ond pi by immunoelectron microscopy for HIF-1α revealed that this protein was mostly localized in the sub-membranous compartment as well as within variably sized, round, moderately to highly electrondense vesicles (Figure 5).

Based on the assumptions that many viruses have been shown to induce an increased production of CD63^+^ exosomes [58], and that viral proteins can be stored within endolysosomal system [59], DL-IF for HIF-1α in association with different markers was performed and evaluated by laser scanning confocal microscopy.

To verify the specificity of the membranous staining, DL-IF for HIF-1α in association with WGA was performed, confirming a membranous to sub-membranous localization of HIF-1α without overlapping co-staining of the two markers (Supplementary Figure S2).

**Figure 6:**
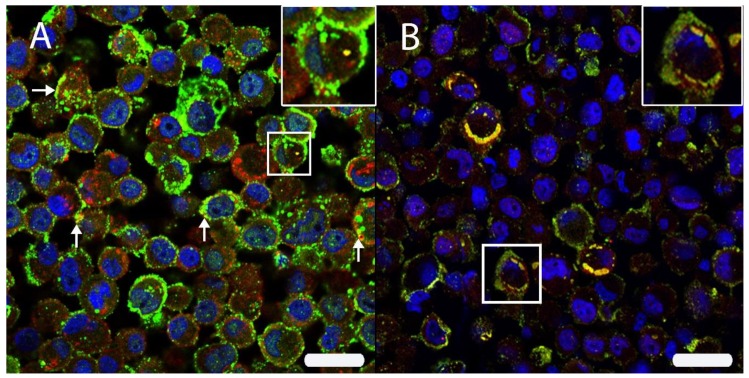
(**A**) The intracellular HIF-1α localization was analyzed by double immunofluorescence with HIF-1α (Cy2, green) and CD63 (Cy3, red) in persistently canine distemper virus (CDV)-infected DH82 cells. Both proteins were localized within cell membranes and cytoplasm. Interestingly, an occasional co-expression (yellow) was noted (arrows; insert) using scanning confocal laser microscopy. (**B**) A double labeling directed against HIF-1α (Cy3, red) and the CDV nucleoprotein (CDV-NP; Cy2, green) revealed a frequent co-localization (yellow) beneath the cell membrane and within the perinuclear area (insert) of persistently CDV-infected DH82 cells. Nuclei were stained with bisbenzimide (blue). Bar = 20 µm.

To investigate whether HIF-1α was associated with exosomes, DL-IF in association with CD63 was performed, revealing an occasional co-localization of the two markers (Figure 6).

To exclude an HIF-1α storage within the Golgi apparatus, DL-IF in association with GM-130 was performed, clearly showing that HIF-1α was not localized within this cell organelle (Supplementary Figure S2).

Finally, to analyze whether HIF-1α was associated with CDV-NP, DL-IF in association with CDV-NP was performed, revealing a marked and diffuse co-localization of the two markers (Figure 6).

In summary, these results confirmed an unexpected localization of HIF-1α in the sub-membranous compartment of DH82Ond pi cells, being occasionally associated with CD63^+^ exosomes and more frequently with CDV-NP. To investigate if this unusual localization of HIF-1α can affect the expression of its angiogenetic downstream molecules with a special focus on VEGF-B, further microarray data and immunofluorescence analyses were performed.

### 3.4. Unexpected Intracellular HIF-1α Localisation Is Associated with a Dysregulated Expression of Angiogenetic Downstream Targets

Among the gene symbols referring to the functional group “HIF-1α angiogenic downstream molecules”, six out of 45 genes were up-regulated, whereas 11 genes were down-regulated (Table 2). Specifically, down-regulated gene symbols included those related to the expression of angiogenetic and anti-angiogenetic macromolecules which transcription is directly induced by the activation of the HIF-1α downstream pathway (i.e. vascular endothelial growth factor B—*VEGFB*; thrombospondin 2—*THBS2*; endothelin 1—*EDN1/ET1*; serine peptidase inhibitor *E—SERPINE1*; thrombospondin 1—*THBS1*; chemokine ligand 12—*Cxcl12*; CD73—*NT5E*; basic fibroblast growth factor 2—*FGF2*, adrenomedullin—*ADM*; *CD274*).

**Figure 7:**
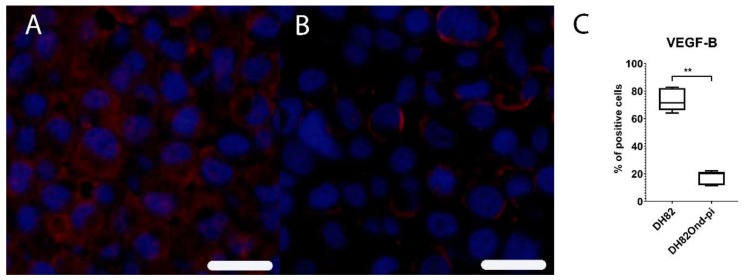
Immunofluorescence analysis for vascular endothelial growth factor B (VEGF-B, Cy3, red) revealed a high expression of this marker in non-infected DH82 cells (**A**), whereas a low expression was present in persistently canine distemper virus (CDV)-infected DH82 cells (**B**); Bar = 20 µm. This statistically significant difference is graphically shown in (**C**). Box and whisker plots display median and quartiles with maximum and minimum values. Significant differences (*p* ≤ 0.05, Mann–Whitney U-test) are labeled by asterisks (** *p* ≤ 0.01).

Immunofluorescence for VEGF-B revealed a significantly (*p* = 0.0079) decreased percentage of immunopositive cells in DH82Ond pi pellets (median = 20.17%, range: 11.52%–22.18%) (Supplementary Table S1) compared to non-infected DH82 pellets (median = 71.41%, range: 64.00%–82.76%), as shown in Figure 7.

Taken together, these results are indicative of a reduced activation of the HIF-1α angiogenic downstream pathway. This is most likely due to an excessive, unusually localized, and non-functional protein expression of HIF-1α, which might be the consequence of a decrease in its cytoplasmic degradation following a virus-induced increased oxidative stress.

## 4. Discussion

Canine histiocytic sarcoma cells (DH82) persistently infected with CDV-Ond display a complete spontaneous tumor regression when xenotransplanted subcutaneously into *Scid* mice [11]. Considered that DH82Ond pi cells did not show any difference in growth and apoptotic rate compared to non-infected controls in vitro and during the initial phase after transplantation in vivo [11,20,21], it was assumed that tumor regression of DH82Ond pi xenotransplants was not caused primarily by direct virus-induced cell death alone. Indeed, it seems more likely that secondary effects of the viral infection on the tumor microenvironment [8,23], as similarly reported for Reoviruses [24], account for the complete regression. Specifically, it was estimated that regression of DH82Ond pi xenotransplants might be related to alterations in cancer-associated angiogenesis [11]. Therefore, the aim of the present in vitro study was to investigate in more detail pathways potentially involved in this regression process, taking advantage of the absence of the confounding effects correlated with ongoing tumor cell death associated with acute CDV-Ond infection [12]. Furthermore, to restrict the complex interactions that occur within a living organism, a less complex, highly standardized in vitro model is assumed to facilitate the analysis of specific intracellular pathways. Interestingly, the so-called “angiogenic switch” has been reported to be one of the most important hallmarks of cancer [22,60], thus playing a central role for tumor development and expansion. In this context, the present study focused on pathways correlated with increased levels of intracellular ROS. These highly reactive molecules have been reported both as fundamental intermediates in physiological intracellular signaling transduction [29,30], as well as in the regulation of different cancer hallmarks [38,45,61]. Specifically, together with hypoxia, ROS represent one of the major activators of HIF-1α [39,40,42,45,61], a transcription factor involved in the regulation of a wide plethora of cancer features such as invasion, metastasis, and angiogenesis [22,38,39,40,41,61]. In the context of the aforementioned considerations, the present study was further directed to investigate the impact of a persistent CDV-Ond infection of DH82 cells on cellular oxidative stress.

CDV has been reported as being able to trigger an increase in ROS intracellular levels, with the subsequent induction of oxidative stress in different kinds of cells such as microglia, in vitro as well as in vivo [31,32,33,34,35]. Similarly, the present study revealed increased ROS levels in DH82Ond pi cells, as demonstrated by an increased oxidative burst, as well as suggested by increased gene transcription of *TXNIP* and *NCF4*. Specifically, the upregulation of both genes might correlate with an increased intracellular oxidative stress. Indeed, *NCF4* encodes for p40^phox^, a protein that is involved in NADPH oxidase 2 activation [29,41,52]. Additionally, thioredoxin-binding protein 2, encoded by the *TXNIP* gene, is an important inhibitor of the thioredoxin ROS scavenging system [29,53]. On the other hand, ROS-induced nucleic acid damage did not differ in DH82Ond pi cells compared to non-infected controls. This observation might be interpreted as indicative of an increased oxidative stress associated with the neoplastic nature of DH82 cells rather than an effect of the viral infection. Similarly, increased intracellular ROS levels are described in the literature as a common feature of cancer cells [38,45,61]. In addition, DH82Ond pi cells displayed an increased expression of SOD2 and CAT compared to non-infected controls. The overexpression of these scavenging enzymes involved in ROS detoxification have been correlated with an increased oxidative stress in neoplastic [38,45,61] as well as in inflammatory conditions [62].

The results obtained by microarray analysis of genes correlated with ER stress [29,31,36,37,45] are consistent with a reduced transcription of genes correlated with this process. The data in the present study might be interpreted as suggestive of an acquired ability of DH82 cells to adapt to the persistent infection with CDV-Ond. However, a marked protein overexpression of ER-stress markers such as calnexin, calreticulin and CHOP/GADD 153 have been observed in Vero cell and primary rat neurons 36 h post-infection with recombinant A75/17-V CDV [31]. On the other hand, the aforementioned lack of differences in growth and apoptotic rate between non infected and DH82Ond pi cells [20,21] is in line with the hypothesis that a persistent infection with CDV-Ond might be associated with the activation of adaptive and pro-survival pathways to contrast prolonged oxidative stress, as reported in recombinant HeLa cells expressing silkworm storage protein 1 [54]. The hypothesis of the present study is further supported by the finding of an increased expression of ROS-scavenging enzymes in DH82Ond pi cells at both a molecular and protein level, highlighting the plasticity of cancer cells in actively contrasting excessively severe alterations in their redox potential [45,61].

The expression of HIF-1α was subsequently investigated due to the observation that increased oxidative stress is associated with an increased HIF-1α stabilization and activation [39,40,42,45,61]. Hypoxia has been widely reported as the most powerful inductor of HIF-1α transcriptional activity [39,40,42]; however, in the present study, cells were cultivated under normoxic conditions. Therefore, hypoxia could be excluded as the cause of the increased HIF-1α protein expression observed in our in vitro model. Consequently, it seems more plausible that the increased expression of HIF-1α in DH82Ond pi cells was induced by the increased oxidative stress level compared to non-infected controls. The down-regulation of 2 PHDs as well as of VHL on a molecular level, in association with a lacking regulation of HIF-1α opposed to an increased expression of the corresponding protein, could imply that the increased protein expression of HIF-1 α in DH82Ond pi cells does not refer to an increased synthesis, but rather to an inhibition of the degradation pathway.

Correspondingly, ROS have been reported to be directly involved in the inhibition of the aforementioned cytoplasmic enzymes (i.e., PHDs and VHL) responsible for HIF-1α hydroxylation and ubiquitination which prelude the rapid degradation of HIF-1α itself by the proteasome 26s [39,40,42].

In addition to the overall increased expression of HIF-1α, the present study revealed an unusual localization of the transcription factor in the sub-membranous compartment and, to a lesser extent, within cytosolic vesicles. Further investigations aiming to better characterize the aforementioned vesicles, revealed a co-localization of HIF-1α expression with CD63, a marker for the tetraspanin-30 expressed by exosomal membranes [63]. Interestingly, the presence of HIF-1α within CD63^+^ exosomes has previously been reported in Epstein-Barr virus-infected NP69 cells [58]. On the other hand, HIF-1α only occasionally co-localized with CD63^+^ exosomes, while it frequently overlapped with the localization of CDV-NP. The measles virus N-protein, which is closely related to CDV-NP [64], is transported within the cell through the endolysosomal system [59], also rendering this a possible mechanism for the canine counterpart. Furthermore, this observation displays an interesting basis for future investigations on the exact sub-cellular localization of HIF-1α within DH82Ond pi cells.

Microarray data analysis aiming to investigate the molecular consequences of the unusual localization of HIF-1α and a prospective loss of function of its transcriptional activity, revealed a significant down-regulation of different genes involved in the HIF-1α angiogenic downstream pathway, which was further substantiated by a significantly reduced expression of VEGF-B on a molecular and protein level. Though VEGF-B is nowadays recognized as not being directly involved in angiogenesis, this growth factor has been reported as an indirect enhancer of VEGF-A (a well-known inducer of angiogenesis), as well as a key promoter of survival of different cell types (including endothelial cells, pericytes and smooth muscle cells) in several pathological conditions [65,66,67]. As already reported in the literature [20], the markedly reduced expression of VEGF-B in DH82Ond pi cells did not affect cellular growth nor the apoptotic rate [21]. Interestingly, DH82Ond pi cell xenotransplants displayed a significantly reduced microvessel density compared to non-infected controls [11]. According to the results of the present study, it can be assumed that HIF-1α might represent an important mediator of the oncolytic effects described for the in vivo model of DH82Ond pi xenotransplants as reported previously in another viral oncolysis model [68].

## 5. Conclusions

Summarized, the results of the current in vitro study are indicative of a reduced activation of the HIF-1α angiogenic downstream pathway in DH82 cells persistently infected with CDV-Ond compared to non-infected controls. This is most likely due to an excessive, unusually localized, and non-functional expression of HIF-1α, which might be the consequence of a decreased cytosolic degradation of this transcriptional factor following a virus-induced increased oxidative stress. Future studies are warranted to better characterize the localization of HIF-1α and the exosomes in which it is contained, as well as to verify the presence of an increased oxidative stress and an aberrant HIF-1α localization in DH82Ond pi also in vivo. The latter approach might further substantiate the assumed correlation between reduced angiogenesis, hypoxia and tumor regression in DH82Ond pi xenotransplants.

## Figures and Tables

**Table 1:** Details of the antibodies used for the immunostaining performed, including primary antibody, host species, clonality, epitope retrieval method, blocking serum, dilution of primary antibody, secondary antibody and positive control.

Primary Antibody	Host Species, Clonality	Epitope Retrieval	Serum Blocking	Dilution	Secondary Antibody (1:200)	Positive Control
CDV-NP (University of Bern)	Mouse, monoclonal, clone D110	Citrate buffer, microwave (800 W, 20´)	PBST + 3% BSA + 5% goat serum	1:100	GaM-Cy3 or GaM-Cy2	n/a
8OHdG (Abcam, Cambridge, USA)	Goat, polyclonal	Proteinase K	PBST + 3% BSA + 5% horse serum	1:200	DaG-Cy3	Canine pyo-granu-lomatous endo- metritis
SOD2 (Abcam, Cambridge, USA)	Rabbit, polyclonal	Citrate buffer, microwave (800 W, 20´)	PBST + 3% BSA + 5% goat serum	1:200	GaR-Cy2	Canine brain and spinal cord
CAT (Abcam, Cambridge, USA)	Goat, polyclonal	Citrate buffer, microwave (800 W, 20´)	PBST + 3% BSA + 5% horse serum	1:50	DaG-Cy3	Canine spinal cord with fibro-carti- lagineous embolus
HIF-1α (Novus Biologicals, Colorado, USA)	Rabbit, polyclonal	Citrate buffer, microwave (800 W, 20´)	PBST + 3% BSA + 5% goat serum	1:500	GaR-Cy3 or DaR-Cy2	Canine mammary adeno- carcinoma with central necrosis
Wheat germ agglutinin (WGA) AF633 conjugated (Invitrogen, California, USA)	none	Citrate buffer, microwave (800 W, 20´)	n/a	1:20	n/a	n/a
CD63 (Sicgen, Coimbra, Portugal)	Goat, polyclonal	Citrate buffer, microwave (800 W, 20´)	PBST + 3% BSA + 5% horse serum	1:200	DaG-Cy3	MDCK cell pellet
GM-130 (BD Transduction Laboratories, North Carolina, USA)	Mouse, monoclonal, clone 35/GM130 (RUO)	Citrate buffer, microwave (800 W, 20´)	PBST + 3% BSA + 5% goat serum	1:200	GaM-Cy2	n/a
VEGF-B (My Biosource, California, USA)	Rabbit, polyclonal	Citrate buffer, microwave (800 W, 20´)	PBST + 3% BSA + 5% goat serum	1:40	GaR-Cy3	Canine fetal brain, liver and kidney

BSA, bovine serum albumin; CDV-NP, canine distemper virus nucleoprotein; DaG-Cy3, donkey anti goat cyanine 3-conjugated; DaR-Cy2, donkey anti rabbit cyanine 2-conjugated; GaM-Cy2, goat anti mouse cyanine 2-conjugated; GaM-Cy3, goat anti mouse cyanine 3-conjugated; GaR-Cy2, goat anti rabbit cyanine 2-conjugated; GaR-Cy3, goat anti rabbit cyanine 3-conjugated; GM130, Golgi membrane protein of 130 kDa; HIF-1α, hypoxia-inducible factor 1 α; MDCK, Madin-Darby canine kidney cells; n/a, non applied or non applicable; PBST, phosphate buffered saline Tween-20; SOD2, superoxide dismutase 2; VEGF-B, vascular endothelial growth factor-B; WGA, wheat germ agglutinin; 8OHdG, 8-hydroxyguanosine/8-hydroxydeoxyguanosine.

**Table 2:** Summary of canine gene symbols related to ROS production and scavenging, ER-stress and HIF-1α pathway, differentially expressed between non-infected and persistently canine distemper virus infected DH82 cells, according to the combination of a fold change (FC) filter (FC ≥ 1.5 or ≤ −1.5) and of a statistical significances filter (*p* ≤ 0.05).

Canine Gene Symbol	Gene Name	Functional Group	Fold Change	*p*-Value	References
***VEGF-B***	vascular endothelial growth factor B	HIF-1a downstream	−593.197	<0.001	[29,39,40,41,45]
***THBS2***	thrombospondin 2	HIF-1a downstream	−451.295	<0.001	[42]
***EDN1***	endothelin 1	HIF-1a downstream	−47.795	<0.001	[42]
***CXCR4***	chemokine (C-X-C motif) receptor 4	HIF-1a downstream	−13.485	<0.001	[39]
***SERPINE1***	serine (or cysteine) peptidase inhibitor, clade E, member 1	HIF-1a downstream	−13.116	<0.001	[41,42]
***COX7B2***	cytochrome c oxidase subunit VIIb2	ROS production; ER stress	−6.015	<0.001	[29,31,45]
***ITPR3***	inositol 1,4,5-triphosphate receptor, type 3	ER stress	−4.646	<0.001	[37]
***THBS1***	thrombospondin 1	HIF-1a downstream	−4.461	<0.001	[42]
***ERO1L***	ERO1-like (*S. cerevisiae*)	ROS production; ER stress	−3.995	<0.001	[37]
***Cxcl12***	chemokine (C-X-C motif) ligand 12	HIF-1a downstream	−3.683	<0.001	[39]
***NT5E***	5’-nucleotidase, ecto (CD73)	HIF-1a downstream	−3.041	<0.001	[39]
***CANX***	calnexin	ER stress	−2.780	<0.001	[36]
***TXNRD3***	thioredoxin reductase 3	ROS scavenging	−2.464	<0.001	[29,45]
***NDUFAF2***	NADH dehydrogenase (ubiquinone) 1 alpha subcomplex, assembly factor 2	ROS production; ER stress	−2.292	<0.001	[29,45]
***NDUFAB1***	NADH dehydrogenase (ubiquinone) 1, alpha/beta subcomplex, 1, 8kDa	ROS production; ER stress	−2.261	<0.001	[29,45]
***DDIT3***	DNA-damage-inducible transcript 3	ER stress	−2.087	<0.001	[36]
***EGLN1***	Egl nine homolog 1 (*C. elegans*)	HIF-1a transcription & regulation	−1.976	0.001	[39,40,42,46]
***PRDX6***	peroxiredoxin 6	ROS scavenging	−1.895	<0.001	[29,45]
***EGLN3***	egl nine homolog 3 (*C. elegans*)	HIF-1a transcription & regulation	−1.875	0.004	[39,40,42,46]
***SDHD***	succinate dehydrogenase complex, subunit D, integral membrane protein	ROS production; ER stress	−1.857	<0.001	[29,45]
***FGF2***	fibroblast growth factor 2 (basic)	HIF-1a downstream	−1.842	0.003	[42]
***PDIA6***	protein disulfide isomerase family A, member 6	ROS production; ER stress	−1.801	<0.001	[37]
***VHL***	von Hippel-Lindau tumor suppressor, E3 ubiquitin protein ligase	HIF-1a transcription & regulation	−1.771	0.005	[39,40,42,45,46]
***SOD1***	superoxide dismutase 1, soluble	ROS scavenging	−1.712	<0.001	[29,45]
***PDIA4***	protein disulfide isomerase family A, member 4	ROS production; ER stress	−1.678	0.010	[37]
***ADM***	adrenomedullin	HIF-1a downstream	−1.665	<0.001	[42]
***GSS***	glutathione synthetase	ROS scavenging; ER stress	−1.648	0.001	[37]
***NDUFC2***	NADH dehydrogenase (ubiquinone) 1, subcomplex unknown, 2, 14.5kDa	ROS production; ER stress	−1.630	0.001	[29,45]
***GCLM***	glutamate-cysteine ligase, modifier subunit	ROS scavenging; ER stress	−1.565	<0.001	[37]
***PDIA3***	protein disulfide isomerase family A, member 3	ROS production; ER stress	−1.533	0.001	[37]
***CD274***	CD274 molecule	HIF-1a downstream	−1.515	0.025	[39]
***PDGFRL***	platelet-derived growth factor receptor-like	HIF-1a downstream	1.554	0.004	[39]
***UQCR11***	ubiquinol-cytochrome c reductase (6.4kD) subunit	ROS production; ER stress	1.563	0.002	[29,45]
***UQCRC2***	ubiquinol cytochrome c reductase core protein 2	ROS production; ER stress	1.590	0.021	[29,45]
***NDUFS1***	NADH dehydrogenase (ubiquinone) Fe-S protein 1, 75kDa (NADH-coenzyme Q reductase)	ROS production; ER stress	1.622	<0.001	[29,45]
***NCF2***	neutrophil cytosolic factor 2	ROS production	1.639	0.004	[29]
***UQCRC1***	ubiquinol-cytochrome c reductase core protein 1	ROS production; ER stress	1.678	<0.001	[29,45]
***ITPR1***	inositol 1,4,5-triphosphate receptor, type 1	ER stress	1.844	0.001	[37]
***NDUFS7***	NADH dehydrogenase (ubiquinone) Fe-S protein 7, 20kDa (NADH-coenzyme Q reductase)	ROS production; ER stress	1.846	<0.001	[29,45]
***LONP1***	lon peptidase 1, mitochondrial	ER stress	1.850	0.001	[37]
***CCL2***	chemokine (C-C motif) ligand 2	HIF-1a downstream	1.866	<0.001	[29,42]
***HMOX1***	heme oxygenase (decycling) 1	ROS scavenging	1.940	<0.001	[29]
***NDUFA10***	NADH dehydrogenase (ubiquinone) 1 alpha subcomplex, 10, 42kDa	ROS production; ER stress	2.009	<0.001	[29,45]
***PDGFA***	platelet-derived growth factor alpha polypeptide	HIF-1a downstream	2.089	<0.001	[39]
***PPID***	peptidylprolyl isomerase D (cyclophilin D)	ER stress	2.286	<0.001	[29,45]
***NDUFV3***	NADH dehydrogenase (ubiquinone) flavoprotein 3	ROS production; ER stress	2.362	<0.001	[29,45]
***ALOX5AP***	arachidonate 5-lipoxygenase-activating protein	ROS production	2.509	<0.001	[45]
***COX17***	COX17 homolog, cytochrome c oxidase assembly protein	ROS production; ER stress	2.557	0.001	[29,31,45]
***CAT***	Catalase	ROS scavenging	3.584	<0.001	[29,45]
***NQO1***	NAD(P)H dehydrogenase, quinone 1	ROS scavenging	3.868	<0.001	[29]
***XDH***	xanthine dehydrogenase	ROS production; ER stress	3.913	0.002	[29,37,45]
***KITLG***	KIT ligand	HIF-1a downstream	4.174	<0.001	[39]
***LOC100856470***	peroxiredoxin-2-like	ROS scavenging	5.351	<0.001	[29,45]
***TEK***	endothelial-specific receptor tyrosine kinase	HIF-1a downstream	5.639	<0.001	[39,41,42]
***PDGFC***	platelet derived growth factor C	HIF-1a downstream	6.578	<0.001	[39]
***TXNIP***	thioredoxin interacting protein	ROS scavenging	11.227	0.001	[29]
***NCF4***	neutrophil cytosolic factor 4, 40kDa	ROS production	67.304	<0.001	[29,41]

Green labeling refers to down-regulated genes; red refers to up-regulated genes. ER, endoplasmic reticulum; HIF-1α, hypoxia-inducible factor 1α; ROS, reactive oxygen species. “HIF-1α transcription & regulation” is the abbreviation for “HIF-1α activation, transcriptional activity and regulation” functional group; “HIF-1α downstream” is the abbreviation for “HIF-1α angiogenic downstream pathway” functional group.

## Supplementary Materials

The following are available online at https://www.mdpi.com/1999-4915/12/2/200/s1, Supplementary Figure S1. Non-infected DH82 cells (A) lacked a canine distemper virus (CDV) specific signal using immunofluorescence for CDV nucleoprotein (CDV-NP, Cy3, red) whereas nearly all cells (median 99.65%, range 99.05–100.00%) express CDV-NP in persistently infected pellets (B). Nuclei were labeled with bisbenzimide (blue). Bar = 20µm, Supplementary Figure S2. Determination of oxidative burst by fluorescence activated cell sorting (FACS) in non-infected (A, B) and persistently canine distemper virus (CDV)-infected (C, D) DH82 cells. For quantification of the percentage of positive cells, doublets were excluded by FCS-A versus FSC-H gating (B, D) and only FL-1-positive cells (Gate 2) of all singlet cells (Gate 1) were quantified, Supplementary Figure S3. (A) The intracellular localization of HIF-1α (Cy2, green) in persistently canine distemper virus (CDV)-infected DH82 cells was analyzed by double immunofluorescence with the cell membrane marker wheat germ agglutinin (WGA, Cy3, red). Furthermore, a double labeling of HIF-1α (Cy3, red) and the golgi matrix protein GM-130 (Cy2, green) was performed in persistently CDV-infected DH82 cells (B). Scanning confocal laser microscopy revealed a membranous co-localization (arrows) for HIF-1α with the cell membrane (A). In contrast, no co-localization was present for HIF-1α and the golgi matrix protein GM-130, excluding the Golgi localization of the protein within the cell (B). Nuclei were stained with bisbenzimide (blue). Bar = 20µm, Supplementary Table S1. Summary of statistical analyses depicting median and mean percentage of immunopositive cells for each cell population (i.e. non-infected and DH82Ond pi cells) or for each specific intracellular localization (i.e., membrane, cytoplasm, or nucleus), with the corresponding minimum-maximum range and standard deviation for each marker investigated. The normality of distribution of each data set as well as the *p*-value of multiple and/or pairwise comparisons between the groups are also reported. Legend: CDV-NP, canine distemper virus nucleoprotein; HIF-1α, hypoxia-inducible factor 1 α; KW, Kruskall-Wallis test, min-max, minimum-maximum range; n/a, not applied or not applicable; SD, standard deviation; SOD2, superoxide dismutase 2; VEGF-B, vascular endothelial growth factor-B; 8OHdG, 8-hydroxyguanosine/8-hydroxydeoxyguanosine; (*), *p* ≤ 0.05; (**), *p* ≤ 0.01, Supplementary Table S2. List of manually selected gene symbols related to ROS production and scavenging, ER-stress- and HIF-1α pathway, with corresponding fold change and *p*-value. Gene symbols significantly down- or up-regulated are highlighted in green and red, respectively. “HIF-1α transcription & regulation” is the abbreviation for “HIF-1α activation, transcriptional activity and regulation” functional group; “HIF-1α downstream” is the abbreviation for “HIF-1α angiogenic downstream pathway” functional group. Complete bibliographic references can be found in the dedicated section within the main manuscript file, numbered as follows: Attig et al. 2019 [31], Bhandary et al. 2013 [37], Brunner et al. 2012 [36], Galadari et al. 2017 [45], Klaunig et al. 2010 [46], Krock et al. 2011 [42], Mittal et al. 2014 [29], Semenza 2014 [39], Ushio-Fukai & Nakamura 2008 [41], Zepeda et al. 2013 [40].
