# Supplementary material for: Oxidative Stress in Canine Histiocytic Sarcoma Cells Induced by an Infection with Canine Distemper Virus Led to a Dysregulation of HIF-1α Downstream Pathway Resulting in a Reduced Expression of VEGF-B In Vitro
Source: Viruses. 2020 Feb 11;12(2):200. doi: 10.3390/v12020200 (PMC7077254; doi:10.3390/v12020200)
Supplement: Supplementary file 1 [file viruses-12-00200-s001.zip › viruses-685782-for conversion-suppl_/viruses-685782-Supplementary_Files_MODIFIED/viruses-685782-Supplementary_Material-File.pdf]

viruses

Oxidative stress in canine histiocytic sarcoma cells induced by an infection with canine distemper virus led to a dysregulation of HIF-1α downstream pathway resulting in a reduced expression of VEGF-B *in vitro*

Federico Armando, Matteo Gambini, Attilio Corradi, Chiara Giudice, Vanessa Maria Pfankuche, Graham Brogden, Friederike Attig, Maren von Köckritz-Blickwede, Wolfgang Baumgärtner, and Christina Puff

Supplementary material:

This file includes:

Figures: 1 to 3

Tables: 1 to 2

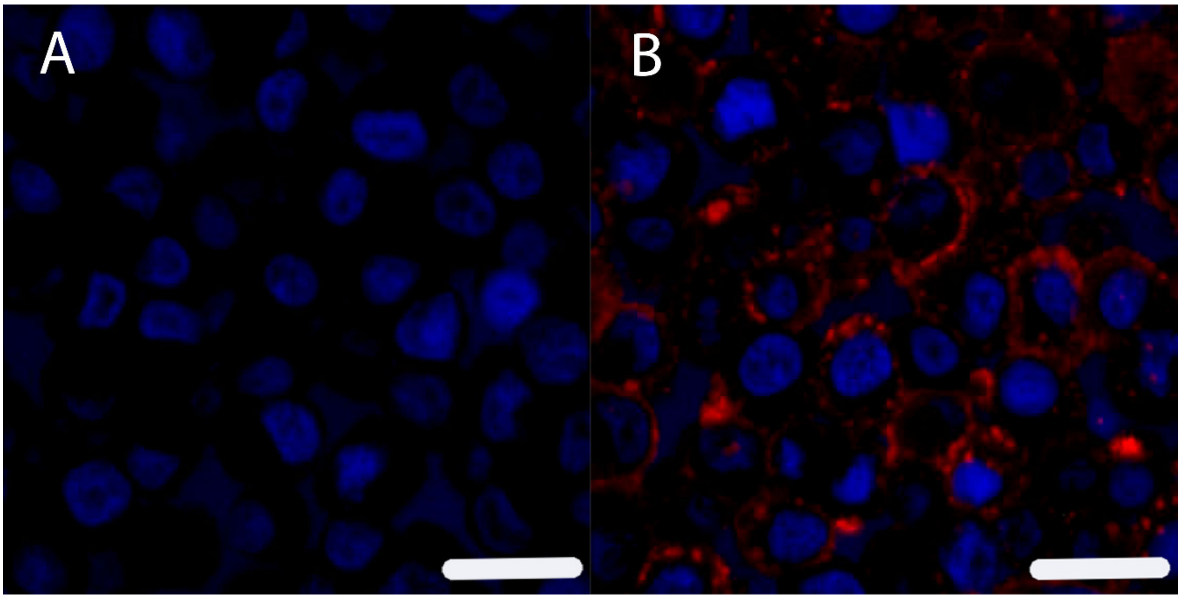

Supplementary Figure 1. Non-infected DH82 cells **(A)** lacked a canine distemper virus (CDV) specific signal using immunofluorescence for CDV nucleoprotein (CDV-NP, Cy3, red) whereas nearly all cells (median 99.65%, range 99.05-100.00%) express CDV-NP in persistently infected pellets **(B)**. Nuclei were labeled with bisbenzimide (blue). Bar = 20µm

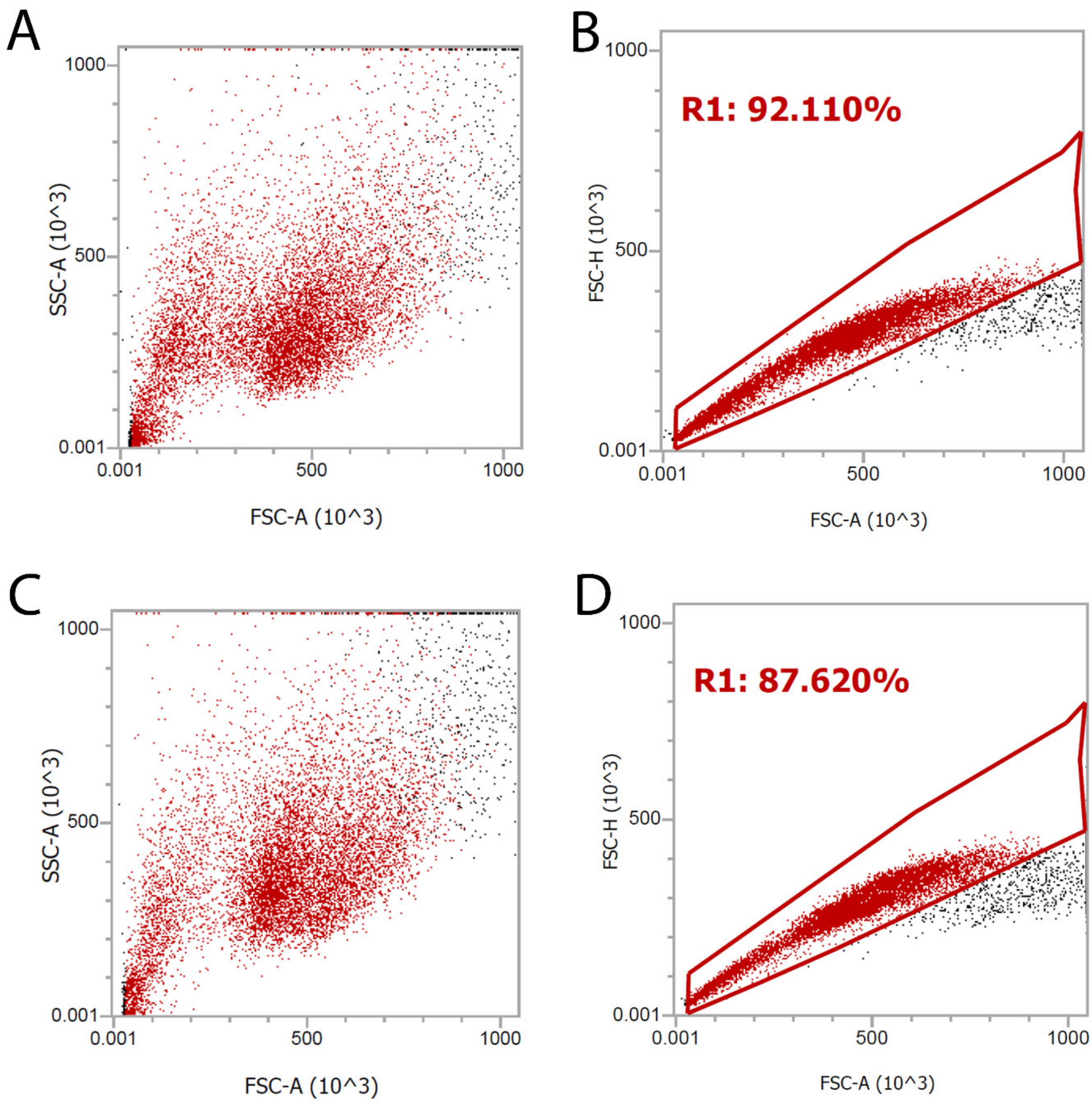

Supplementary Figure 2. Determination of oxidative burst by fluorescence activated cells sorting (FACS) in non-infected **(A, B)** and persistently canine distemper virus (CDV)-infected **(C, D)** DH82 cells. For quantification of the percentage of positive cells, doublets were excluded by FCS-A versus FSC-H gating **(B, D)** and only FL-1-positive cells (Gate 2) of all singlet cells (Gate 1) were quantified.

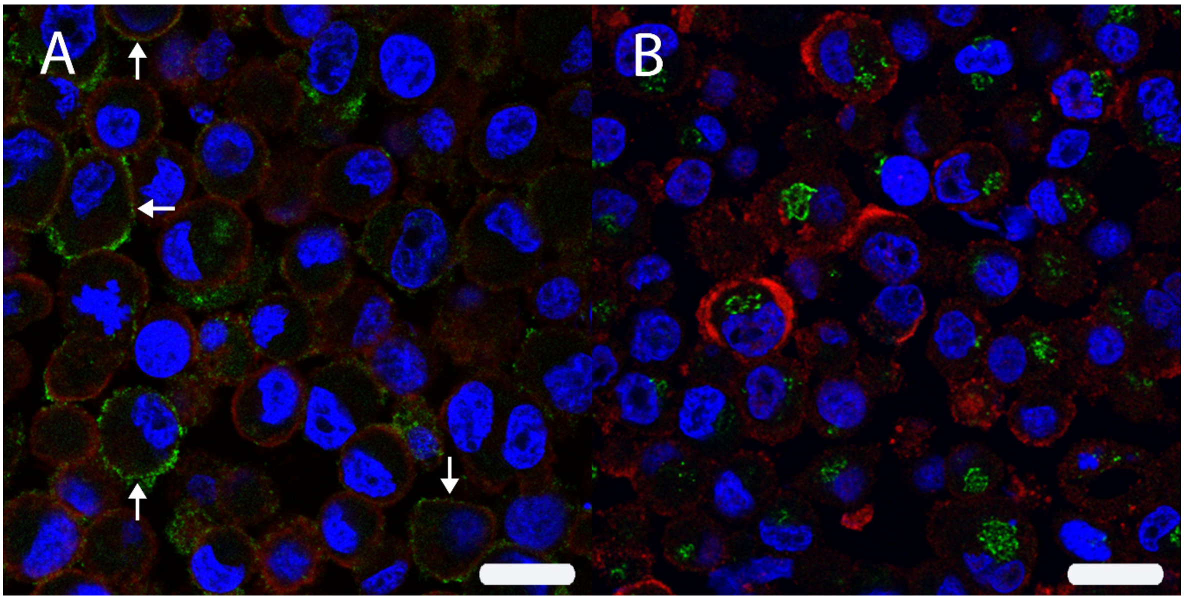

Supplementary Figure 3. **(A)** The intracellular localization of HIF-1α (Cy2, green) in persistently canine distemper virus (CDV)-infected DH82 cells was analyzed by double immunofluorescence with the cell membrane marker wheat germ agglutinin (WGA, Cy3, red). Furthermore a double labeling of HIF-1α (Cy3, red) and the golgi matrix protein GM-130 (Cy2, green) was performed in persistently CDV-infected DH82 cells **(B)**. Scanning confocal laser microscopy revealed a membranous co-localization (arrows) for HIF-1α with the cell membrane **(A)**. In contrast, no co-localization was present for HIF-1α and the golgi matrix protein GM-130, excluding the Golgi localization of the protein within the cell **(B)**. Nuclei were stained with bisbenzimide (blue). Bar = 20μm

Supplementary Table 1. Summary of statistical analyses depicting median and mean percentage of immunopositive cells for each cell population (i.e. non infected and DH82Ond pi cells) or for each specific intracellular localization (i.e. membrane, cytoplasm, or nucleus), with the corresponding minimum-maximum range and standard deviation for each marker investigated. The normality of distribution of each data set as well as the *p*-value of multiple and/or pairwise comparisons between the groups are also reported.

Marker	Group	Median (%)	Range (min-max)	Mean (%)	SD	Normality (Shapiro-Wilk test)	Mann-Whitney U-test	Kruskall-Wallis test + Dunn's test
**CDV-NP**	DH82	0.00	0.00-0.00	0.00	0.00	n/a	n/a	n/a
	DH82Ond-pi	99.65	99.05-100.00	99.60	0.3825	Yes		
**8OHdG**	DH82	96.80	94.58-100.00	97.26	2.070	Yes	P = 0.5476	n/a
	DH82Ond-pi	99.33	95.94-99.79	98.57	1.637	Yes		
**SOD2**	DH82	0.00	0.00-0.47	0.09	0.21	No	p = 0.0079 (**)	n/a
	DH82Ond-pi	20.39	7.75-27.30	16.93	8.72	Yes		
**Catalase**	DH82	37.27	19.61-39.94	31.63	10.05	Yes	p = 0.0079 (**)	n/a
	DH82Ond-pi	81.29	72.92-90.58	82.24	6.56	Yes		
**HIF-1α**	DH82	2.53	2.24-9.51	4.46	3.16	Yes	p = 0.0079 (**)	n/a
	DH82Ond-pi	36.95	28.83-39.99	34.50	5.21	Yes		
**HIF-1α distribution (DH82)**	A) Membrane	20.75	0.00-35.94	18.22	14.20	Yes	n/a	• KW p=0.1708<br>• A)-B) p=0.5373<br>• A)-C) p=0.1980<br>• B)-C) p>0.9999
	B) Cytoplasm	30.38	20.31-95.24	41.89	30.98	No		
	C) Nucleus	43.69	4.76-69.49	39.89	23.15	Yes		

Supplementary Table 1 cont.

Marker	Group	Median (%)	Range (min-max)	Mean (%)	SD	Normality (Shapiro-Wilk test)	Mann-Whitney U-test	Kruskall-Wallis test<br>• + Dunn's test
**HIF-1α distribution (DH82Ond-pi)**	A) Membrane	64.74	22.80-85.02	58.08	24.41	Yes	n/a	• KW p=0.0176 (*)<br>• A)-B) p=0.0710<br>• A)-C) p=0.0486 (*)<br>• B)-C) p>0.9999
	B) Cytoplasm	21.01	10.78-25.58	18.25	6.26	Yes		
	C) Nucleus	14.06	4.20-29.05	15.63	10.86	Yes		
**HIF-1α MEMBRANE**	DH82	20.75	0.00-35.94	18.22	14.20	Yes	p=0.0317 (*)	n/a
	DH82Ond-pi	64.74	22.80-85.02	58.08	24.41	Yes		
**HIF-1α CYTOPLASM**	DH82	30.38	20.31-95.24	41.89	30.98	No	p=0.0952	n/a
	DH82Ond-pi	21.01	10.78-25.58	18.25	6.26	Yes		
**HIF-1α NUCLEUS**	DH82	43.69	4.76-69.49	39.89	23.15	Yes	p=0.0952	n/a
	DH82Ond-pi	14.06	4.20-29.05	15.63	10.86	Yes		
**VEGF-B**	DH82	71.41	64.00-82.76	73.57	8.24	Yes	p=0.0079 (**)	n/a
	DH82Ond-pi	20.17	11.52-22.18	17.33	5.16	Yes		

Legend: CDV-NP, canine distemper virus nucleoprotein; DH82, non-infected DH82 cells; DH82Ond pi, persistently CDV-infected DH82 cells; HIF-1α, hypoxia-inducible factor 1 α; KW, Kruskall-Wallis test; min-max, minimum-maximum range; n/a, not applied or not applicable; SD, standard deviation; SOD2, superoxide dismutase 2; VEGF-B, vascular endothelial growth factor-B; 8OHdG, 8-hydroxyguanosine/8-hydroxydeoxyguanosine; (*), $p \leq 0.05$; (**), $p \leq 0.01$.

Supplementary Table 2. List of manually-selected gene symbols related to ROS production and scavenging, ER-stress- and HIF-1α pathway, with corresponding fold change and p-value. Gene symbols significantly down- or up-regulated are highlighted in green and red, respectively. "HIF-1α transcription & regulation" is the abbreviation for "HIF-1α activation, transcriptional activity and regulation" functional group; "HIF-1α downstream" is the abbreviation for "HIF-1α angiogenic downstream pathway" functional group. Complete bibliographic references can be found in the dedicated section within the main manuscript file, numbered as follows: Attig et al. 2019 [31], Bhandary et al. 2013 [37], Brunner et al. 2012 [36], Galadari et al. 2017 [45], Klaunig et al. 2010 [46], Krock et al. 2011 [42], Mittal et al. 2014 [29], Semenza 2014 [39], Ushio-Fukai & Nakamura 2008 [41], Zepeda et al. 2013 [40].

Supplementary Table 2 is submitted as a separate MS Office Excel file
