# Supplementary figures and images for: Oxidative Stress in Canine Histiocytic Sarcoma Cells Induced by an Infection with Canine Distemper Virus Led to a Dysregulation of HIF-1α Downstream Pathway Resulting in a Reduced Expression of VEGF-B In Vitro
Source: Viruses. 2020 Feb 11;12(2):200. doi: 10.3390/v12020200 (PMC7077254; doi:10.3390/v12020200)

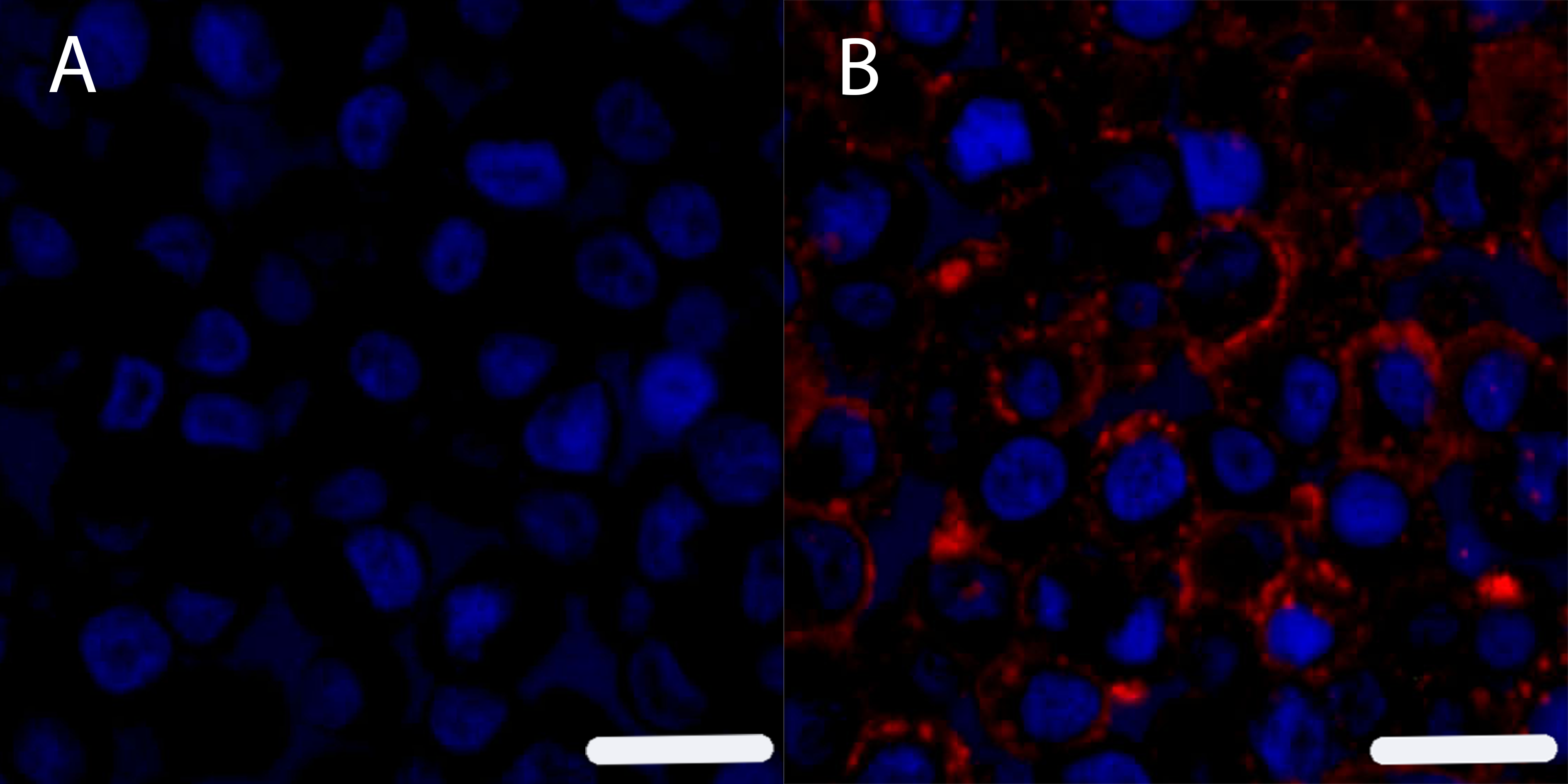

Supplement: Supplementary file 1 [file viruses-12-00200-s001.zip › viruses-685782-for conversion-suppl_/viruses-685782-Supplementary_Files_MODIFIED/viruses-685782-Supplementary_Figure_1.tif]

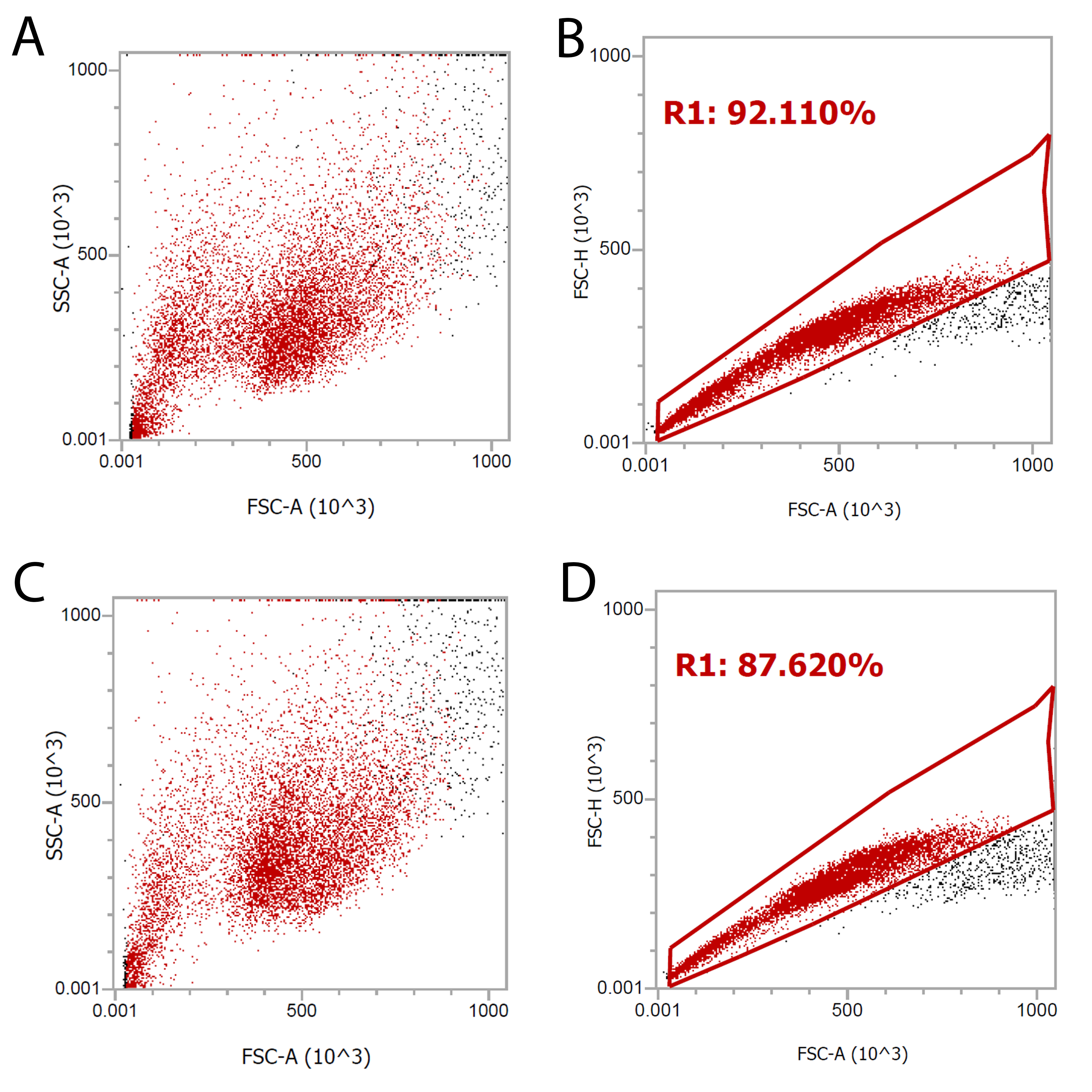

Supplement: Supplementary file 1 [file viruses-12-00200-s001.zip › viruses-685782-for conversion-suppl_/viruses-685782-Supplementary_Files_MODIFIED/viruses-685782-Supplementary_Figure_2.tif]

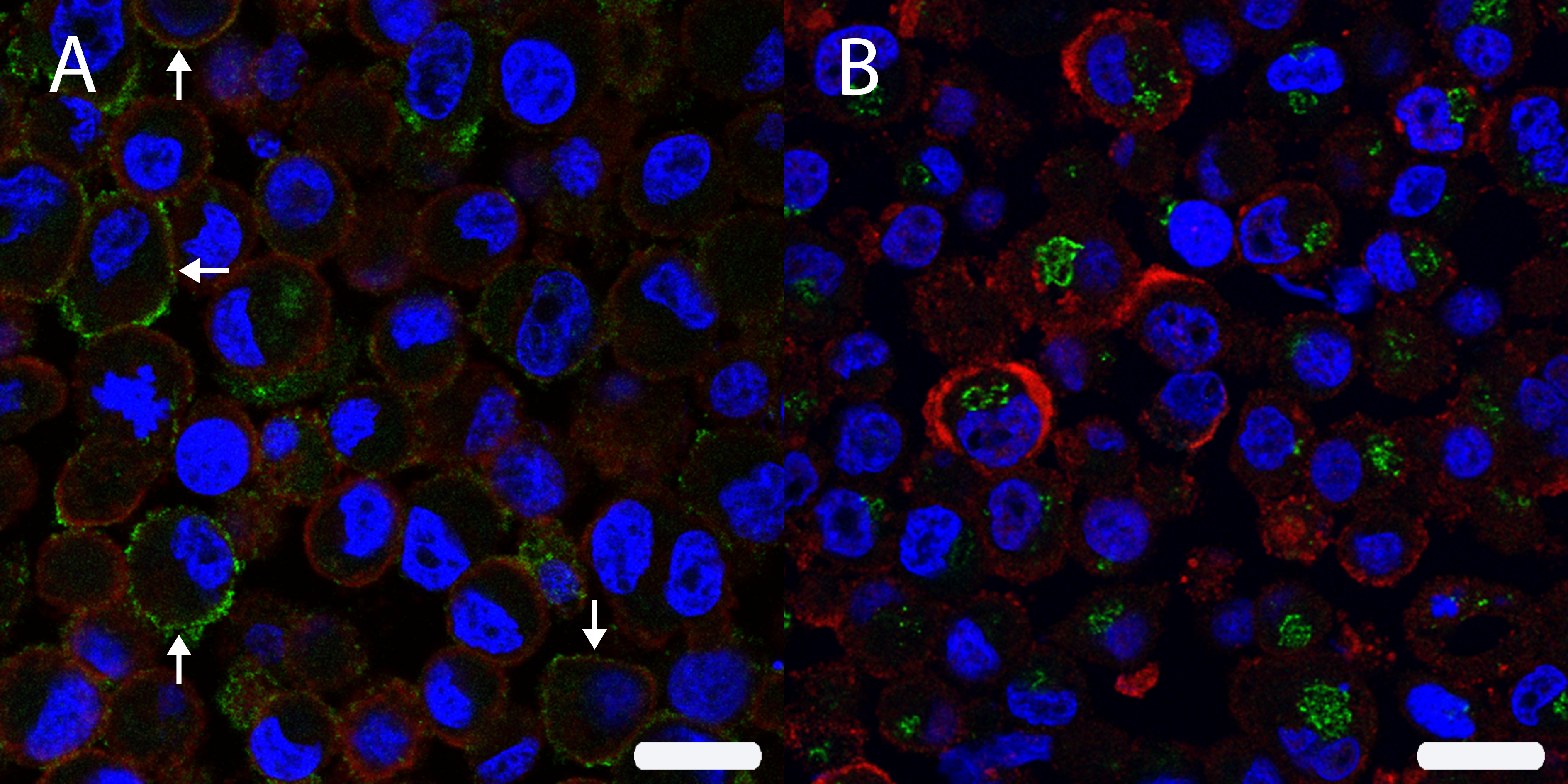

Supplement: Supplementary file 1 [file viruses-12-00200-s001.zip › viruses-685782-for conversion-suppl_/viruses-685782-Supplementary_Files_MODIFIED/viruses-685782-Supplementary_Figure_3.tif]
